# Culture-Negative Infective Endocarditis Post-mitral Valve Repair: A Case Illustrating the Role of Advanced Imaging in Surgical Decision-Making

**DOI:** 10.7759/cureus.94746

**Published:** 2025-10-16

**Authors:** Kayla West, Margaret Ho, Pristine Floyde, Alexander Kraev, K. Dean Gubler, Shunsuke Aoi

**Affiliations:** 1 Emergency Medicine, Rocky Vista University College of Osteopathic Medicine, Englewood, USA; 2 Radiology, Brooke Army Medical Center, San Antonio, USA; 3 Emergency Medicine, Rocky Vista University College of Osteopathic Medicine, Billings, USA; 4 Thoracic Surgery/Cardiothoracic Surgery, Billings Clinic, Billings, USA; 5 Surgery, Rocky Vista University College of Osteopathic Medicine, Ivans, USA; 6 Interventional Cardiology, Billings Clinic, Billings, USA

**Keywords:** advanced cardiac imaging, blood culture negative endocarditis, cardiac computed tomography (cct), cardiothoracic & vascular surgery research, infective endocarditis, prosthetic valve infection, surgical mitral valve repair, transthoracic and transesophageal echocardiography

## Abstract

Endocarditis is a rare but serious condition that can occur in patients with valvular heart disease, particularly after valvular repair or in those with prosthetic heart valves. While it is generally considered less common following successful valvular repairs without evidence of active infection, endocarditis can present in a variety of ways, including as an incidental finding in patients with stroke. Stroke in the setting of endocarditis is often caused by embolic events originating from infected valve leaflets or vegetations. Although blood cultures remain the gold standard for diagnosing infective endocarditis, negative blood cultures are not uncommon, particularly in early or indolent cases. Advanced imaging modalities, such as transesophageal echocardiography (TEE) and cardiac computed tomography (CT), play a crucial role in identifying valve abnormalities, vegetations, and other structural heart lesions that may suggest endocarditis, especially when blood cultures fail to provide a definitive diagnosis. This case report focuses on the diagnostic challenges and the role of advanced imaging in a patient with a history of prior mitral valve repair who presented with recurrent stroke events but was otherwise asymptomatic. Despite initial negative blood cultures, suggestive of early or indolent endocarditis, serial imaging with cardiac CT and TEE guided clinical decision-making and timely intervention. Dynamic changes in echogenic structures over time prompted surgical intervention before microbiologic confirmation, underscoring the importance of imaging in prosthetic valve patients who may present atypically, such as with stroke as the sole manifestation. In addition, the study explores whether stroke can be the sole presenting symptom of endocarditis in patients with valvular disease or repair. The case further emphasizes the need for high suspicion of endocarditis in patients with valvular heart disease, especially those with embolic events, and underscores the importance of timely imaging to guide management decisions. This case also provides a review of similar reports in the literature, reinforcing the role of cardiac CT and TEE in diagnosing endocarditis in patients with valvular disease and prosthetic valve repairs.

## Introduction

This article was previously presented as a poster at the 2025 Association of Military Osteopathic Physicians and Surgeons on February 20, 2025. Infective endocarditis (IE) remains a life-threatening disease with high morbidity and mortality, particularly in patients with predisposing factors such as prosthetic heart valves, congenital heart disease, prior valvular repair, or intravenous drug use [1]. Despite advances in medical and surgical management, diagnosing IE remains challenging, especially when traditional clinical criteria are inconclusive. The modified Duke criteria, which incorporate clinical, microbiologic, and imaging findings, serve as the primary diagnostic tool but have notable limitations in prosthetic valve disease and culture-negative infections [2]. This challenge is particularly relevant in patients with prior mitral valve repair, where residual valvular changes or non-infectious thrombotic material can complicate imaging interpretation.

One of the most concerning complications of IE is embolic stroke, occurring in up to 20-40% of cases [3]. Stroke in the context of IE is typically caused by septic embolization of valvular vegetations to the cerebral vasculature. While embolic stroke is often accompanied by systemic signs of infection, such as fever and leukocytosis, some patients present with cerebrovascular events as their initial or sole manifestation. This atypical presentation can delay diagnosis, particularly when blood cultures remain negative. Culture-negative endocarditis, accounting for approximately 10% of cases, presents an additional diagnostic hurdle and is often due to prior antibiotic exposure or infection with fastidious organisms that are difficult to isolate [2,4]. Given the potential for devastating neurologic outcomes, prompt recognition and early intervention are critical in patients with suspected embolic stroke secondary to IE.

In recent years, advanced imaging modalities have become increasingly vital in evaluating endocarditis, particularly when standard diagnostic criteria are inconclusive. Techniques such as transthoracic echocardiography (TTE), transesophageal echocardiography (TEE), and cardiac computed tomography (CT) are commonly used; however, in patients with prior mitral valve repair or prosthetic valves, differentiating infectious vegetations from non-infectious findings like pannus or thrombus remains challenging [5,6]. In these cases, supplementary imaging tools are often necessary to enhance diagnostic accuracy and guide clinical decision-making.

This case underscores the complexities of diagnosing infective endocarditis in a patient with an atypical presentation. It highlights the challenges posed by negative blood cultures and the absence of classic infectious signs, emphasizing the critical role of advanced imaging in guiding clinical management. Additionally, it explores the importance of maintaining a high index of suspicion for endocarditis in patients with embolic stroke and a history of valvular intervention. This report discusses diagnostic challenges, the role of multimodal imaging, and implications for clinical care in such cases.

## Case presentation

**Figure 1:**
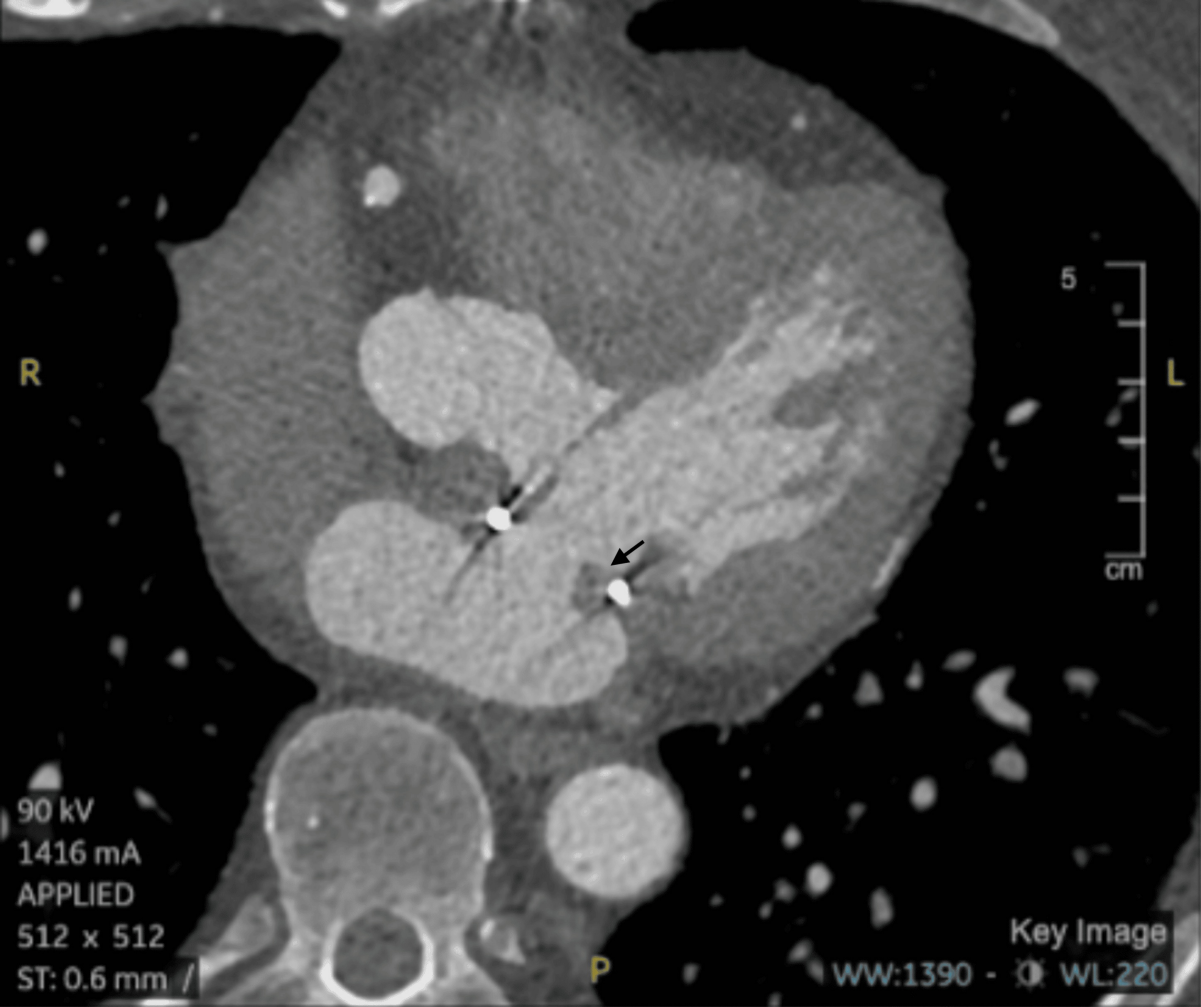
Cardiac Computed Tomography Imaging of the Prosthetic Mitral Valve Axial computed tomography image demonstrating abnormality around the prosthetic mitral valve repair, including early pannus formation and possible thrombus or vegetation. Image was obtained from the patient with their permission.

**Figure 2:**
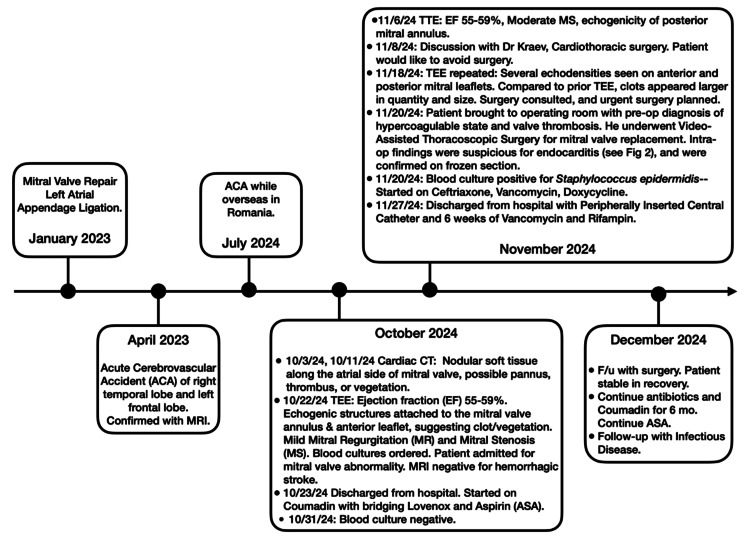
Timeline of Medical Events Chronological overview of the patient’s cerebrovascular events, imaging studies, and interventions related to suspected infective endocarditis. Key dates include initial stroke (April 2023), recurrent stroke (July 2024), cardiac CT and transesophageal echocardiography (TEE) evaluations, follow-up imaging, and eventual surgical intervention. Figure Credits: Kayla West, primary author.

A 63-year-old male with a history of hypertension, obstructive sleep apnea, dyslipidemia, and mitral valve repair in 2023 presented to his primary care physician with multiple presyncopal episodes, several months after experiencing his second cerebral infarction in July 2024, following a prior stroke in April 2023. Given his recurrent embolic events and history of mitral valve repair, his primary care physician ordered a cardiac CT with and without contrast to evaluate for potential cardiac sources of embolism. Notably, additional risk factors included a recent dental procedure, poor dental hygiene, recent colonoscopy, with no history of injection drug use or other immunocompromising conditions. A few possible etiologies under consideration included IE, thrombus formation in the setting of a hypercoagulable state, pannus overgrowth, and cardioembolic stroke secondary to arrhythmia. The CT identified abnormal soft tissue around the prosthetic mitral annulus but was unable to definitively differentiate between pannus, thrombus, or vegetation (see Figure 1). Due to the uncertainty, a TEE was recommended for further evaluation (see Figure 2 for timeline of events).

**Figure 3:**
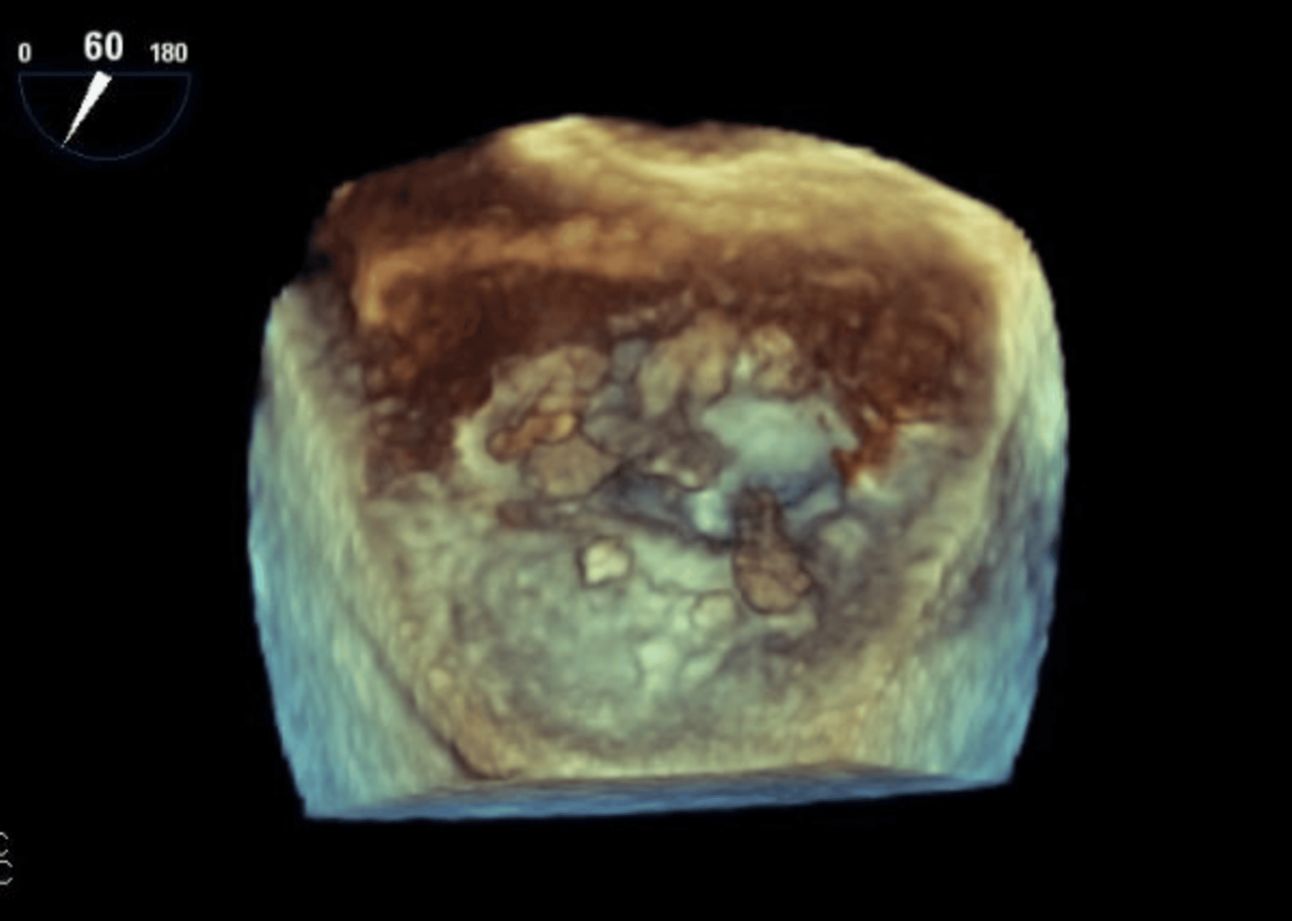
Transesophageal Echocardiography (TEE) of the Prosthetic Mitral Valve TEE images showing echogenic structures attached to the atrial aspect of the anterior and posterior mitral leaflets, concerning for thrombus or vegetation. Image was obtained from the patient with their permission.

The patient underwent a TEE 11 days later, which identified an echogenic structure attached to the mitral annulus and anterior leaflet, concerning for either clot or vegetation (see Figure 3). Left ventricular function was preserved, with an ejection fraction of 55-59% with moderate mitral stenosis and mild tricuspid regurgitation. Given these findings and the patient's history of embolic strokes, his cardiologist recommended urgent evaluation in the emergency department for further workup, including blood cultures and a brain magnetic resonance imaging (MRI) to assess for any new infarcts. The MRI revealed chronic lacunar infarcts, bilateral thalami and left cerebellum with no acute infarcts. Additionally, three sets of blood cultures on 10/22/2024 remained negative at that time. 

A previously scheduled TTE was performed during his admission, showing findings similar to those noted on TEE: confirmed echodensity, immobile structure suggestive of either chronic clot/scar or healed vegetation. Given the diagnostic uncertainty and concern for potential endocarditis versus noninfectious thrombotic material, the case was discussed with the cardiothoracic surgery team. After extensive discussion, the patient opted to defer immediate surgical intervention and instead follow up with a repeat TEE in approximately one month to assess for interval changes. 

The follow-up TEE demonstrated several new echodensities attached to the atrial aspect of both the anterior and posterior mitral leaflets. Compared to his prior study, the lesions had increased in size and number, raising further concern for an ongoing pathological process. Importantly, the patient’s initial blood cultures were negative, consistent with early, culture-negative endocarditis. Serial imaging over the ensuing weeks demonstrated enlarging echodensities on the mitral valve, highlighting dynamic changes in valve pathology. Given the high risk of embolization and mortality associated with potential clot dislodgment, these progressive imaging findings - rather than laboratory confirmation alone - were the primary driver of the decision to proceed with mitral valve replacement. After further discussions with his cardiologist and cardiothoracic surgery team, he consented to the procedure.

**Figure 4:**
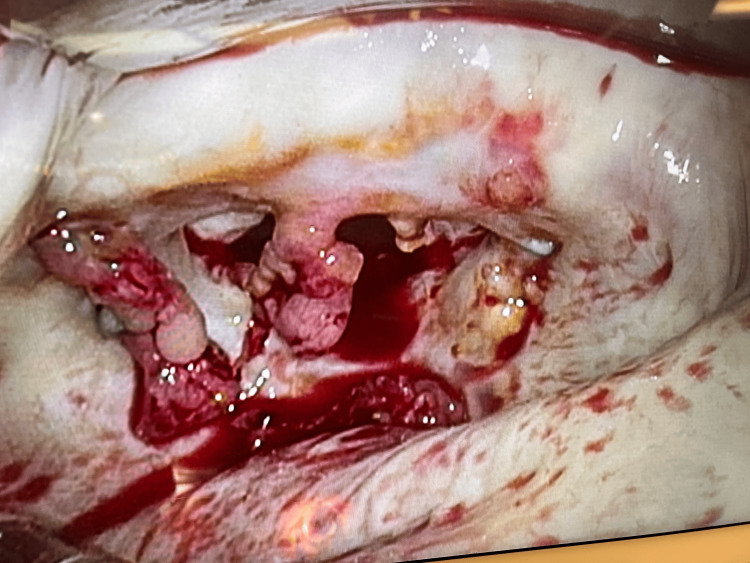
Endocarditis Vegetations Intraoperative view of the mitral valve showing friable vegetations consistent with infective endocarditis. Image obtained from the patient’s surgical record and used with permission; adapted for this article.

Following surgical intervention, intraoperative findings confirmed friable material on the mitral valve leaflets, and subsequent blood cultures several days later returned positive for *Staphylococcus epidermidis *(see Figure 4). Pathologic examination of the intraoperative biopsy was consistent with infective endocarditis caused by *Staphylococcus epidermidis*. This case highlights the challenges in diagnosing culture-negative endocarditis and underscores the importance of multimodal imaging and a high index of suspicion in patients with recurrent embolic strokes and prior valvular intervention. 

## Discussion

This case illustrates an atypical presentation of IE in a patient with a history of mitral valve repair. Unlike classical cases of *Staphylococcus epidermidis* IE, which typically present with fever, leukocytosis, and positive blood cultures, this patient was afebrile, hemodynamically stable, and had persistently negative blood cultures. Notably, the patient did not initially meet the modified Duke criteria for definitive IE: three major criteria, one major and three minor, or five minor criteria (see Table 1) [2]. At the time of initial evaluation, only two of the five minor clinical criteria were satisfied, including predisposing valvular disease and embolic stroke, with standard CT imaging unable to definitively identify the presenting abnormality on the initial valve repair. 

**Table 1:** Duke Criteria to Diagnose Infective Endocarditis (IE) Modified Duke Criteria for the Diagnosis of Infective Endocarditis. Table constructed by the primary author (Kayla West). Content adapted from the Modified Duke Criteria as described in [2]. This table was independently created and does not reproduce copyrighted material.

Category	Criteria
Major	Blood Culture Positive
Major	Evidence of IE
Minor	Predisposing Heart Condition
Minor	Fever
Minor	Vascular Phenomenon
Minor	Immunologic Phenomenon
Minor	Microbiologic Evidence
Pathologic	Microorganism in a vegetation
Pathologic	Pathologic Lesion

The modified Duke criteria, while widely used, have reduced sensitivity in cases involving prosthetic valves and culture-negative infections. This patient’s eventual diagnosis was only established postoperatively, after intraoperative pathology and valve cultures confirmed *S. epidermidis* infection. With modern, stringent surgical precautions, *S. epidermidis* is an unusual pathogen for culture-negative IE. This highlights a critical diagnostic gap, as culture-negative endocarditis constitutes up to 10% of IE cases and often necessitates reliance on clinical suspicion and advanced imaging [7]. Its identification in this case highlights the potential for the persistence of indolent infections in prosthetic material, particularly in the absence of systemic symptoms. 

**Figure 5:**
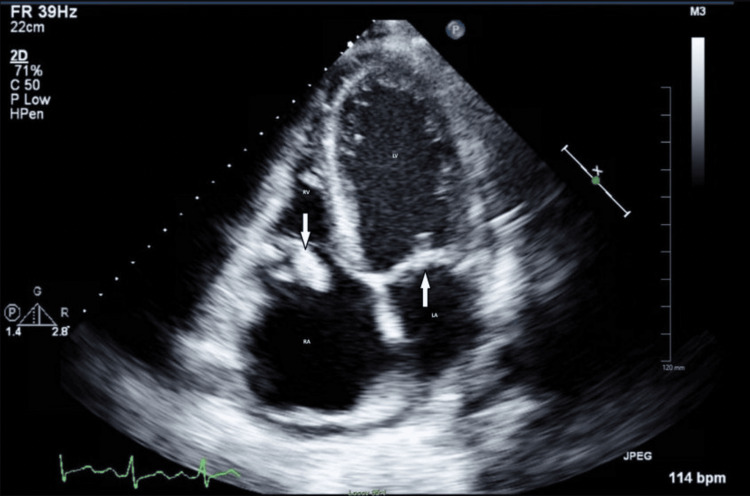
Transthoracic Echocardiography (TTE) of the Tricuspid and Mitral Valve Representative TTE image illustrating tricuspid and mitral vegetations noted on apical four-chamber view. Image reproduced with permission from the original publisher [8].

The role of advanced imaging modalities cannot be overstated, especially when standard diagnostic criteria are inconclusive. While clinical evaluation and history provided initial context, imaging modalities such as CT, TEE, and TTE were pivotal in diagnosing and guiding management. Each modality offers unique strengths and limitations in assessing prosthetic valve endocarditis, necessitating careful selection based on clinical suspicion and diagnostic yield (see Table 2) [4,5]. Figures 1, 3 show the patient’s actual cardiac scans and images, while Figure 5 presents a representative example image for comparison [8]. Initial CT imaging provided a broad overview, detecting abnormality of the prosthetic valve, but lacked the resolution to differentiate between pannus, thrombus, or vegetation. Initial TTE and TEE identified echogenic structures attached to the mitral annulus and anterior leaflet; however, they were unable to differentiate between clot and vegetation. It was ultimately a follow-up TEE that demonstrated progressive and newly developing echodensities of the atrial aspect of both anterior and posterior mitral leaflets that led to escalation of care, surgical intervention, and eventual diagnosis of culture-negative IE despite the absence of major Duke criteria and most of the minor criteria. This finding emphasizes the critical importance of repeated imaging in difficult cases and suggests that dynamic changes over time may serve as an important clinical marker, particularly when microbiologic data is inconclusive. TEE remains the gold standard for evaluating prosthetic valve endocarditis due to its superior sensitivity and resolution compared to TTE. Cardiac CT serves as a valuable adjunct, particularly for detecting complications or for evaluating surrounding structures, but cannot replace the role of TEE in suspected IE. 

**Table 2:** Diagnostic Imaging Comparison Chart for Infective Endocarditis in Prosthetic Valve Repairs Comparison of sensitivity, specificity, utility, invasiveness, image quality, and limitations of imaging modalities (computed tomography (CT), transthoracic echocardiogram (TTE), transesophageal echocardiogram (TEE)) in prosthetic valve endocarditis [4,5]. Table constructed by the primary author (Kayla West).

Characteristic	CT	TTE	TEE
Sensitivity	~64%	~70%	~92-96%
Specificity	~88%	~91-98%	~95-100%
Utility in Diagnosis	Used primarily for detecting complications (abscesses, septic emboli, paravalvular extension) and structural anomalies	First-line test for screening, helpful for assessing valve motion and vegetations	Gold standard for detecting vegetations, abscesses, and valve perforations
Invasiveness	Non-invasive	Non-invasive	Invasive (requires sedation)
Image Quality	High-resolution, can identify emboli, abscesses, paravalvular extension and other complications	Good for vegetations, but less clear for small lesions or abscesses	High-quality, excellent for visualizing small vegetations and valve abnormalities
Limitations	Limited in detecting vegetations on the valves, but excellent for abscesses and emboli	Can miss small vegetations or abscesses, less effective in obese patients	Requires expertise, not always available in emergency settings

Emerging diagnostic approaches, such as molecular methods (organism-specific polymerase chain reaction (PCR), broad-range 16S rRNA PCR, targeted or shotgun metagenomic sequencing) and radiotracer-based imaging, represent promising tools for the future diagnosis of infective endocarditis [9]. These were not reviewed in depth here, as our focus was on established imaging modalities (CT, TTE, and TEE) most relevant to the clinical course of the present case.

This case also highlights several important teaching points for clinicians managing prosthetic valve patients. First, stroke may be the sole presenting manifestation of endocarditis in patients with prosthetic valves, even in the absence of fever or positive blood cultures. Second, vegetations may be missed initially, especially on standard imaging, necessitating serial and repeated imaging to detect dynamic changes over time. Third, surgical intervention may be required prior to microbiologic confirmation, as progressive imaging findings and risk of embolization can justify early operative management. Finally, culture-negative endocarditis underscores the importance of integrating clinical suspicion, imaging, and patient risk factors rather than relying solely on laboratory confirmation. Collectively, these points reinforce the critical role of dynamic imaging and clinical vigilance in guiding timely intervention for prosthetic valve endocarditis.

## Conclusions

This case of culture-negative IE due to *S. epidermidis* highlights critical diagnostic challenges. The patient’s atypical presentation - absence of systemic infection signs, negative blood cultures, and initial imaging uncertainty - resulted in delayed diagnosis and appropriate care, exposing the limitations of current diagnostic frameworks such as the Duke criteria, particularly in patients with prosthetic valves or prior valvular repairs. This patient’s eventual diagnosis was heavily reliant on serial imaging, which reinforces the importance of maintaining a high suspicion for IE in patients who present with embolic phenomena and a history of prosthetic material, regardless of blood culture results.

Furthermore, this case demonstrates the benefits of the adjunction of varying imaging modalities: TTE and cardiac CT for initial screening and anatomic clarification, and TEE for high-resolution detection of valvular pathology. Timely serial imaging - alongside multidisciplinary collaboration - was paramount in achieving the correct diagnosis and guiding effective treatment. It was diagnostic suspicion by the patient’s care team and early surgical intervention guided by imaging findings, rather than traditional microbiologic criteria alone, that proved to be superior in providing life-saving treatment.

Emerging molecular diagnostic techniques, such as organism-specific PCR and metagenomic sequencing, may in the future provide earlier microbiologic confirmation of infective endocarditis. While not yet widely available due to cost, these methods have the potential to complement imaging and traditional microbiology, improving diagnostic accuracy and enabling earlier intervention.
